# Anabolic-Androgenic Steroids and Exercise Training: Breaking the Myths and Dealing With Better Outcome in Sarcopenia

**DOI:** 10.3389/fphys.2022.838526

**Published:** 2022-03-17

**Authors:** Hugo Falqueto, Marcelo Rodrigues dos Santos, Leandro H. Manfredi

**Affiliations:** ^1^Medical School, Federal University of Fronteira Sul, Chapecó, Brazil; ^2^Graduate Program in Biomedical Sciences, UFFS, Chapecó, Brazil; ^3^Heart Institute (InCor), University of São Paulo Medical School, São Paulo, Brazil

**Keywords:** exercise, anabolic-androgenic steroid, sarcopenia, muscle, resistance trainig

## Abstract

Sarcopenia is an emerging clinical condition determined by the reduction in physical function and muscle mass, being a health concern since it impairs quality of life and survival. Exercise training is a well-known approach to improve physical capacities and body composition, hence managing sarcopenia progression and worsening. However, it may be an ineffective treatment for many elderly with exercise-intolerant conditions. Thus, the use of anabolic-androgenic steroids (AAS) may be a plausible strategy, since these drugs can increase physical function and muscle mass. The decision to initiate AAS treatment should be guided by an evidence-based patient-centric perspective, once the balance between risks and benefits may change depending on the clinical condition coexisting with sarcopenia. This mini-review points out a critical appraisal of evidence and limitation of exercise training and AAS to treat sarcopenia.

## Introduction

The world population continues to age rapidly due to an increase in human life expectancy and a decrease in the birth rate over the past years (Kingston et al., 2018; Chang et al., 2019). This biological process, known as aging, is related to the increase in the incidence of physical disability (Tieland et al., 2018; Suetta et al., 2019; Alcazar et al., 2021a), which contribute to a worse quality of life and greater morbidity and mortality among older adults. The physical function has shown to be negatively associated with mortality than other classical risk factors, such as hypertension, diabetes, smoking, and body mass index (Wu et al., 2017; Yusuf et al., 2020; Whelton et al., 2021). Physical function is a general indicator of functional status and is the manifestation of physical capacities (i.e., muscle strength and power) and performance in functional tests in the daily functional context of older adults (Pavasini et al., 2016; Wu et al., 2017; Tieland et al., 2018; Valenzuela et al., 2019; Alcazar et al., 2021b).

Sarcopenia, a word derived from Greek, means “poverty of flesh,” and it was originally defined as the age-related loss of muscle mass (Clark and Manini, 2008). Currently, sarcopenia is broadly defined as an age-related abnormal loss of skeletal muscle mass and physical function (Cruz-Jentoft et al., 2019; Bhasin et al., 2020b). Sarcopenia progression is multifactorial and complex and it is now recognized as an independently reportable medical condition (ICD-10-CM) (Falcon and Harris-Love, 2017).

According to five cohort studies over 10 years, mortality risk was increased by 49% in those individuals with walking difficulty (a physical function task) (Suemoto et al., 2017). In a prospective cohort study, severe sarcopenia was associated with a 4-fold increase in the risk of death compared to older adults without sarcopenia after 2.6 years of follow-up (Bachettini et al., 2020). This result reveals the high negative impact of this condition in a short period, requiring effective interventions. Approximately 10% of the worldwide population has sarcopenia (Shafiee et al., 2017). However, this estimation can reach or exceed 50% in octogenarian adults who are hospitalized or with some clinical condition (Papadopoulou et al., 2020). Despite different criteria used to diagnose sarcopenia, this condition is strongly associated with increased mortality (Xu et al., 2021).

Sarcopenia can also be associated with pathological processes. Some interventions may revert or attenuate muscle mass loss and physical function, such as exercise. It is known that older adults who are less physically active are more likely to have lower skeletal muscle mass and function, which may lead to an increased risk of developing sarcopenia and, hence, decreasing life expectancy (Brown et al., 2012; Meier and Lee, 2020; Ramsey et al., 2021). Exercise training interventions can be used to restore or maintain physical function in older adults. Moreover, for any given amount of physical activity, mortality risk is lower than a sedentary lifestyle (Brown et al., 2012).

The physiological balance between anabolic and catabolic processes in tissues is altered during aging, especially in those with sarcopenia (Basualto-Alarcón et al., 2014; McKee et al., 2017). Anabolic agents, such as testosterone, have been used in clinical trials with positive results on sexual and physical function in older patients (Snyder et al., 2016; Storer et al., 2017). However, the association of both exercise training and anabolic hormones is a field of intense debate. In a randomized clinical trial (RCT) in patients with heart failure, the association of exercise training and testosterone therapy reversed muscle wasting when compared to testosterone alone (Dos Santos et al., 2016). This combined intervention shows a promising intervention in older adults with sarcopenia and it might be appreciated in clinical practice.

The purpose of this narrative review is to discuss the role of anabolic-androgenic steroids (AAS) and exercise training as a possible treatment of sarcopenia in older adults with sarcopenia.

## Sarcopenia Definition and Diagnosis

Sarcopenia is defined by the loss of physical function and muscle mass (Fielding et al., 2011; Cruz-Jentoft et al., 2019; Bhasin et al., 2020b). The International Working Group on Sarcopenia (IWGS) and European Working Group on Sarcopenia in Older People (EWGSOP) present several screening processes to diagnose sarcopenia. The prevalence of sarcopenia can be different depending on the population, definitions of sarcopenia, body composition measurements, physical function tests, and cutoff point values. For instance, there are different skeletal muscle mass measurements such as relative appendicular skeletal muscle index (RASM) and percentage skeletal muscle index (SMI); and different functional tests: grip strength, chair stand, gait speed, short physical performance battery (SPPB), and the timed-up and go test (TUG). In clinical practice, the use of a simple questionnaire can be helpful to identify individuals at risk for sarcopenia. The SARC-F is a five-item questionnaire that is self-reported by patients as a screen for sarcopenia risk. For more details on screening and diagnosis of sarcopenia, see the guidelines (Fielding et al., 2011; Cruz-Jentoft et al., 2019).

## Does Exercise Prevent or Revert Sarcopenia? Evidence and Limitation of Current Studies

Exercise training is a cornerstone to aid the sarcopenia-related loss of muscle mass and physical function (Fragala et al., 2019; García-Hermoso et al., 2020; Grgic et al., 2020; Izquierdo et al., 2021b). Physical function is determined by multifactorial elements, in which the neuromuscular and metabolic systems play a role in some physical capacities, such as strength, power, agility, and balance (Reid and Fielding, 2012; Tieland et al., 2018; Valenzuela et al., 2019). In addition, other factors, such as lifestyle, psychosocial, and body composition (muscle mass and body fat) may also be variables that may impair physical function (Tieland et al., 2018; Alcazar et al., 2021a). It is well established that exercise promotes changes in the determinants of physical function, hence promoting several benefits to an individual’s overall health (Valenzuela et al., 2019; García-Hermoso et al., 2020; Letnes et al., 2021).

Resistance Training (RT) is considered the most effective type of exercise to improve strength and muscle mass, and consequently, physical function (Fragala et al., 2019; Valenzuela et al., 2019; Grgic et al., 2020; Schaun et al., 2021). Recent guidelines of International Exercise Recommendations for older adults (ICFSR) highlight that RT must be prescribed according to the individual’s need and intended outcome (e.g., promote lifestyle change, improve physical capacities, or disease treatment). In addition, RT must be adjusted, followed by a health specialist, whenever necessary (Izquierdo et al., 2021b). The RT recommendation is based on:

- frequency of 2–3 times a week;
- volume of 1–3 sets of 8–12 repetitions;
- load progression starting at 30%–40% of 1 repetition maximum (1RM) with goals of 70%–80% 1RM.

Exercise can modulate specific physical capacities [e.g., strength, power, and cardiorespiratory fitness (CRF)] and movement demands that simulate the elderly's daily life activities, such as sit-to-stand from a chair, climbing stairs, and lifting objects off the ground (Izquierdo et al., 2021b). Comprehensive exercise recommendations for older adults can be read in different guidelines (Fragala et al., 2019; Izquierdo et al., 2021b).

In sarcopenic elderly people, the use of RT techniques such as a cluster set (additional short intra-set or inter-repetition rest intervals) can minimize fatigue, reduce perceptions of effort, improve exercise tolerance, and increase training volume (Latella et al., 2021). This strategy facilitates the increase in strength, muscle hypertrophy (Borde et al., 2015), power (Carneiro et al., 2020), and physical function (Ramirez-Campillo et al., 2018).

Recently, the multimodal or multicomponent exercise program (MEP) has gained attention not only by improving strength and muscle mass, but also other physical capacities (Courel-Ibáñez et al., 2021a,b; Gonçalves et al., 2021; Izquierdo et al., 2021a). MEP combines different exercise strategies in the same routine and promotes a dynamic and high-adherence activity that aids to improve physical capacities and skills in addition to those classically seen in sarcopenia, such as balance, CRF, and power (Courel-Ibáñez et al., 2021a,b; Izquierdo et al., 2021a). MEP includes exercise strategies, such as (i) high-velocity RT to improve muscle power (Rodriguez-Lopez et al., 2021; Schaun et al., 2021); (ii) high-intensity interval training (HIIT) to improve CRF and cardiovascular risk profile (Blackwell et al., 2021; Letnes et al., 2021); and (iii) balance exercise to improve postural control and reduce the risk of falls (Lesinski et al., 2015; Gerards et al., 2017).

Although the beneficial effects of exercise go beyond increasing muscle function, some patients or even elderly individuals may not benefit from these adaptations due to exercise intolerance (e.g., frailty, cardiorespiratory disability, etc.), severe disability (e.g., bed rest conditions, cachexia conditions, neurological disorders, etc.), adverse or blunted response to exercise, and low adherence (Seynnes et al., 2004; Rivera-Torres et al., 2019; Lalande et al., 2020; Valenzuela et al., 2020b). In addition, many sarcopenic patients may not meet the minimum criteria for exercise recommendations according to recent guidelines (Fragala et al., 2019; Izquierdo et al., 2021b).

A meta-analysis study with 985 sarcopenic participants has demonstrated that exercise significantly increased muscle strength (grip strength, knee extension), physical function (timed up and go, chair-stand, and gait speed), and muscle mass when compared to control (Zhang et al., 2021b). However, another meta-analysis has shown no differences in muscle mass in sarcopenic individuals who were submitted to exercise when compared to control (Hsu et al., 2019; Bao et al., 2020; Escriche-Escuder et al., 2021). These conflicting results can be explained by the different criteria adopted in the inclusion of participants, exercise protocols, and the criteria used in sarcopenia diagnosis. For instance, sarcopenic obese individuals who need to reduce body weight and maintain muscle mass were excluded in some studies (Zhang et al., 2021b), as well as those with several clinical conditions that make adherence to the exercise protocol unfeasible (Balachandran et al., 2014; Piastra et al., 2018; Tsekoura et al., 2018). These aforementioned studies put on the spot that some sarcopenic individuals may not benefit from an exercise program.

Despite the well-known efficacy of physical exercise on the improvement of physical function in older adults, data regarding other important health outcomes are less clear, such as falls, hospitalization length of stay, and mortality. For instance, exercise may not be effective in improving quality of life, risk of hospital admissions, fractures, and mortality in the elderly population (Bhasin et al., 2020a; García-Hermoso et al., 2020; Stensvold et al., 2020; Ballin and Nordström, 2021). Moreover, in acutely hospitalized older adults, exercise does not exhibit clear benefits in the length of stay or the rate of hospital readmission and mortality (Valenzuela et al., 2020a).

In summary, exercise training (together with the nutritional approach) is the best non-pharmacological treatment to prevent or treat sarcopenia. However, older sarcopenic adults, who cannot adhere properly to exercise due to any reason (clinical conditions, physical and phycological impairment, advanced sarcopenia, etc), must have an alternative method to prevent or treat the loss of physical function. Moreover, some evidence calls into question the eligibility of some patients to adhere to adequate exercise protocols, hence beneficial aspects of exercise will not promote changes of important clinical outcomes, such as mortality risk and hospitalization admission.

## Anabolic-Androgenic Steroids When Exercise Seems Ineffective: Evidence and Limitations

Hormonal changes play a fundamental role in the pathophysiology of sarcopenia (Basualto-Alarcón et al., 2014; Shin et al., 2018). Blood concentrations of anabolic hormones are known to decrease as humans age and this may interfere with muscle mass and physical functions (McKee et al., 2017; Stangl et al., 2019). AAS are well-known for their muscle ergogenic and anabolic effects. The AASs comprise the endogenous testosterone and its pharmacology-derivated molecules, in which the chemical structure of testosterone was modified to confer distinct patterns of muscle anabolism and/or androgenic effects (Srinivas-Shankar and Wu, 2006; Kicman, 2008; Hoffman et al., 2009).

It is well-known that a normal physiological decline in anabolic hormones occurs over the years, particularly testosterone. The decline in testosterone plasmatic levels may be intensified with low physical capacities in aging individuals. The Cooper Center longitudinal study has shown that low testosterone levels were directly associated with decreased CRF in elderly men (DeFina et al., 2018). In a cohort study (≥65 years; *n* = 2,587), an inverse association was shown between testosterone levels and physical function and risk of falls in 4 years of follow-up. Those individuals with testosterone levels lower than the 25% quartile reference values exhibited a 40% increase in the risk of falls (Orwoll et al., 2006). In addition, cross-sectional population-based studies have shown that low testosterone levels were associated with decreased muscle strength, poor mobility, decreased muscle mass, and increased risk of falls (Schaap et al., 2005; Chiu et al., 2020). Furthermore, a pronounced testosterone decrease in aging men is associated with high mortality when compared to a normal decline in this hormone in age-matched individuals (Holmboe et al., 2018). Therefore, sarcopenic individuals who present a markedly decrease in testosterone, to lower levels than would be expected, are prone to exhibit more physical function impairment, hence more negative clinical outcomes.

Sex-hormone binding globulin (SHBG), a glycoprotein that binds to sex hormones like testosterone, unlike total and free testosterone, increases with age, hence reducing testosterone bioavailability (Liu et al., 2007; Ramachandran et al., 2019). Due to the increase in SHBG, reductions in bioavailable testosterone show more pronounced declines when compared to total testosterone in aging (Fabbri et al., 2016; Marriott et al., 2021). Recently, the United Kingdom Biobank study demonstrated that high levels of SHBG are a potential biomarker of sarcopenia (Petermann-Rocha et al., 2020). Thus, SHBG appears to play an important role, raising the question of whether or not those individuals with normal levels of total testosterone may present any benefit in the loss of physical function seen during aging.

In a recent review of interventional studies with men and women (≥40 years), exercise training has been shown to provide an increase in the plasmatic anabolic hormones, including testosterone, human growth hormone, insulin-like growth factor-1, and dehydroepiandrosterone sulfate (Zouhal et al., 2021). However, different meta-analyses evaluating exercise interventions have not found an increase in the baseline of total and free testosterone from adults and the elderly (Hayes and Elliott, 2018; Potter et al., 2021). Further studies should be conducted to address the role of exercise training in restoring/maintaining normal hormonal values in the sarcopenic elderly, which could make AAS therapy an interesting approach to improve this condition.

Despite the well-known effects of AAS on increasing muscle mass and physical function, their exogenously use is not a consensus in the management of sarcopenia since these drugs are recognized for their adverse effects (increased levels of hematocrit, blood pressure, cardiovascular and prostate cancer risks, etc.) (Loeb et al., 2017; Gagliano-Jucá and Basaria, 2019; Chasland et al., 2021; Wittert et al., 2021). It is noteworthy that many of these effects are reported by off-label and misuse of testosterone in which supraphysiological plasma levels are chronically sustained for recreational, aesthetic, and muscle-building purposes (Baggish et al., 2017; de Ronde and Smit, 2020; Bhasin et al., 2021a). However, the adverse effects are much less or non-existent in controlled clinical trials, suggesting that AAS could be an approach to sarcopenia management when correctly and safely administered, followed by expertise in the field (Snyder et al., 2018; Diem et al., 2020).

Currently, the most studied and recommend exogenously use of AAS is the Testosterone Replacement Therapy (TRT) for men with hypogonadism (Ponce et al., 2018; Diem et al., 2020). The TRT is used to improve libido, sexual function, and quality of life in symptomatic men with abnormally low blood testosterone levels. However, TRT is not recommended for improving muscle function or increasing muscle mass in this population due to low levels of evidence in the literature (Bhasin et al., 2018b; Corona et al., 2020; Qaseem et al., 2020). Caution should be taken with hypogonadism TRT studies since the recommendations do not apply to treat or manage sarcopenia.

Loss of physical function and muscle mass are associated with physical fatigue, being a common secondary symptom of hypogonadism. However, fatigue *per si* is a subjective complaint and report of these individuals, and physical function is generally not evaluated by health professionals to precisely determine whether fatigue is due to psychosomatic, muscle impairment, sarcopenic status, or another origin. Sarcopenia and hypogonadism may be closely related, despite the prevalence of sarcopenic people with hypogonadism are still unknown (Bhasin et al., 2018b; Corona et al., 2020; Qaseem et al., 2020). Many sarcopenic men may also meet clinical criteria for hypogonadism, and TRT in this case can also improve physical function (Parahiba et al., 2020; Varanoske et al., 2020).

An umbrella review from the Belgian Society of Gerontology and Geriatrics has shown that TRT promoted a strong effect on muscle mass and a modest-to-minimal effect on muscle strength and physical function. In addition, the authors suggest that TRT may be recommended in men with low serum levels of total testosterone (<200–300 ng/dl) to manage the sarcopenia syndrome (De Spiegeleer et al., 2018). In a recent meta-analysis with 773 middle-aged and elderly men, TRT was associated with an increase in muscle mass, strength, and physical performance (Parahiba et al., 2020). Although this study was not conducted exclusively with sarcopenic individuals, the results demonstrated a significant effect of TRT in improving some sarcopenic features (muscle mass and physical function).

In another meta-analysis conducted by Varanoske et al. (2020), TRT *per si* increased lower body, upper body, handgrip strength, lower body muscular endurance, and functional test performance. Functional performance only improved in patients with clinical conditions and older adults (>60 years), but not in younger men (<60 years). In this study, TRT was shown to be more effective in increasing muscle mass than muscle strength or physical function. In addition, TRT can promote a better physical function in sarcopenic than non-sarcopenic older adults. The Testosterone Trials, a double-blind 3-year RCT, have shown that TRT in non-sarcopenic older men was associated with modest, but significant improvement in muscle mass and physical function (Storer et al., 2017). These findings suggest a possible role of TRT in preventing or decreasing the progression of sarcopenia.

Recent evidence has shown that AAS therapy is effective in sarcopenia management (Parahiba et al., 2020; Varanoske et al., 2020). However, there is no clinical consensus that recommends AAS administration in sarcopenia. This is probably due to the lack of RCTs conducted exclusively with sarcopenic patients. In addition, sarcopenia is a condition associated with and/or secondary to other clinical conditions, such as type 2 diabetes mellitus (T2DM), metabolic syndrome (MetS), obesity, anemia, osteoporosis, etc. (Batsis and Villareal, 2018; Bani Hassan et al., 2020; de Freitas et al., 2020; Kirk et al., 2020; Kim et al., 2021a).

It has recently been proposed that the use of AAS (more specifically TRT) should be based on a patient-centered perspective, taking into account the balance between benefit and risk of AAS prescription (Bhasin, 2021; Bhasin and Ozimek, 2021). Thus, the use of AAS can be favored when it is also indicated for other clinical conditions that coexist with sarcopenia. In Table 1, the applicability of using AAS for these conditions is presented according to high-level evidence studies.

**Table 1:** Clinical conditions coexist with sarcopenia and applicability of anabolic androgenic steroids.

Clinical condition	Prevalence in the sarcopenic or older adults	AAS effects	Comments
Hypogonadism	1.5%–12.5% of older men have primary or secondary hypogonadism (Khera et al., 2016).	AAS type: TRT Several consensus and meta-analyses demonstrate that TRT is effective in improving libido and sexual function in men with hypogonadism. Evidence: clinical consensus (Bhasin et al., 2018b; Corona et al., 2020; Qaseem et al., 2020; Salonia et al., 2021).	Hypogonadism may be a secondary condition of sarcopenia. TRT is a well-established therapy in the management of hypogonadism and may benefit sarcopenic patients with hypogonadism to increase muscle mass and physical function.
MetS	~30% in the older adults (Scuteri et al., 2005; Kim et al., 2021a).	AAS type: TRT ↓waist circumference and fat mass; Improvement in glucose metabolism; ↓ HbA1c; ↓ HOMA-IR; ↓ total cholesterol; and Evidence: systematic review and meta-analysis (Corona et al., 2021; Kim et al., 2021b).	TRT may aid in the management of sarcopenia and MetS in older man who need to reduce abdominal fat and improve glycemic and total cholesterol control.
T2DM	~21% of older adults with T2DM are sarcopenic (de Freitas et al., 2020).	AAS type: TRT TRT therapy reduces the proportion of patients with TDM2 associated with lifestyle change when compared to placebo. Evidence: 2-year RCT phase IIIb (*n* = 1,007) (Wittert et al., 2021).	TRT may aid in the management of TDM2 in sarcopenic men aged 50–74 years with pre-TDM2 or newly diagnosed conditions.
Obesity (sarcopenic obesity)	Prevalence of sarcopenic obesity in the elderly population varies depending on the criteria used: Men: 0.1%–85.3% Women: 0%–80.4% (Batsis and Villareal, 2018; Purcell et al., 2021).	AAS type: TRT TRT associated with lifestyle change attenuate the weight loss–induced reduction in muscle mass when compared to placebo. Evidence: 6-month RCT (*n* = 83) (Barnouin et al., 2021).	TRT can attenuate the weight loss–induced reduction in muscle mass that is common in obese people under energy-restricted conditions.
Unexplained Anemia	~7% of the sarcopenic elderly are anemic (Bani Hassan et al., 2020; Tseng et al., 2021).	AAS type: TRT ↑ Hb levels in older men with unexplained anemia and low testosterone when compared to placebo. Evidence: 1-year RCT (*n* = 788) (Roy et al., 2017) AAS type: ND ↑ Hb levels in osteoporotic older women when compared to placebo. Evidence: 2-year RCT (*n* = 65) (Frisoli et al., 2005).	Unexplained anemia can cause fatigue and loss of physical function in older men with low testosterone. TRT increase Hb levels and may explain the improvement in physical function in these individuals. ND may be a therapeutic option for sarcopenic older women with unexplained anemia.
Osteoporosis (Osteosarcopenia)	~5%–37% of osteoporotic patients are sarcopenic (Kirk et al., 2020).	AAS type: ND ↑ lean body mass, ↑ bone mineral density ↓vertebral fractures when compared to placebo in osteoporotic older women. Evidence: 2-year RCT (*n* = 65) (Frisoli et al., 2005) AAS type: TRT ↑ bone mineral density in older men when compared to placebo. Evidence: 1-year RCT (*n* = 211) (Snyder et al., 2017); 2-year RCT (*n* = 601) (Ng Tang Fui et al., 2021).	Osteosarcopenia is considered a clinical entity. AAS can increase bone mineral density while promoting increased muscle mass and physical function. TRT may be an option for older men with low testosterone and ND may be viable for older women as it is a AAS with poor androgenic potential.

AAS, anabolic-androgenic steroids; Hb, *hemoglobin*; HbA1c, glycated haemoglobin; HOMA-IR, homeostasis model assessment of *insulin resistance*; MetS, metabolic syndrome; ND, nandrolone decanoate; RCT, randomized clinical trial; T2DM, type 2 diabetes mellitus; and TRT, testosterone replacement therapy.

Clinical conditions shown in Table 1 are commonly seen in elderly sarcopenic individuals. Then, the use of AAS promotes beneficial outcomes, by increasing not only muscle mass and physical function, but also by improving the clinical parameters related to these conditions.

## A Brief Look at Nutritional and Medication in Sarcopenia

Malnutrition can be defined as inadequate bioavailability of nutrients that lead to decreased physical and mental functions and compromised quality of life and survival (Cederholm et al., 2017). It is considered a predictor of the incidence and prevalence of sarcopenia (Beaudart et al., 2019; Sieber, 2019). Sarcopenic older adults have lower total caloric intake (macronutrients and micronutrients) compared to non-sarcopenic elderly, and this contributes to the state of catabolism and anabolic resistance (Santiago et al., 2021). Sarcopenia associated with malnutrition is a hard-to-treat condition by only administering a correct dietary regimen, since the long-term results of supplementation may not overcome the unbalanced anabolic hormones and the chronic low-grade inflammation seen in these individuals (Reckman et al., 2019). In addition, adding macronutrients to increase the caloric intake can be a barrier for older adults, particularly those with other medical conditions, who naturally reduce their caloric intake, appetite, masticatory, and gastrointestinal functions (Soenen et al., 2016; van Dronkelaar et al., 2019; Senoo et al., 2020).

Nutritional interventions, evaluated in meta-analyses studies, have shown a minimal effect on sarcopenia progression, and a null effect in those groups that perform exercise training (Yoshimura et al., 2017; Choi et al., 2021; Wu et al., 2021). OPTIMen Trial has shown that the amount of protein intake (1.3 vs. 0.8 g/kg/day) did not affect muscle mass and physical function when compared to the groups that used placebo instead of TRT. However, in the TRT groups (testosterone enanthate, 100 mg/week), muscle mass and physical function were increased, regardless of the amount of protein ingested (Bhasin et al., 2018a). These data suggest that restoring testosterone to physiological levels may promote a better outcome in sarcopenia than nutritional approaches *per se*. The effectiveness of nutritional interventions to promote changes in the baseline of testosterone levels is unclear (Henning et al., 2014; Zamir et al., 2021).

As aging progress, different morbidities affect the population, hence the use of medication is a common scenario to treat prevalent diseases. It is estimated that 21% of older adults with T2DM are sarcopenic (de Freitas et al., 2020), and most of them use metformin, the first-line medication to treat this condition (American Diabetes Association, 2021). In the MASTERS trial, metformin has been proved to attenuate muscle hypertrophy in response to resistance training in the elderly population (Walton et al., 2019). On the other hand, the SPRINT trial has demonstrated that exercise training is effective in improving physical function, regardless of whether the participant is a statin and/or antihypertensive user (Alturki et al., 2021).

As shown in Table 1, TRT seems to improve glycemic control and may counteract the deleterious effect of metformin on muscle anabolism in sarcopenic elderly patients with T2DM under metformin treatment. Further studies should be carried out to confirm these findings. Other drugs frequently used by the elderly population to treat cardiovascular disorders do not appear to have a significant effect on sarcopenia (Alturki et al., 2018; Zhang et al., 2021a).

The impact of co-administration of testosterone and other drugs used to treat common age-related diseases needs to be clarified by further investigations to support better interventions that will beneficiate the individual’s health.

## Uncertainties

Although a positive AAS dose–response relation promotes an increase in strength and muscle mass, the administration route seems to play an important role to manage sarcopenia (Bhasin et al., 2001; Borst and Yarrow, 2015). A meta-analysis demonstrated that intramuscular TRT promotes a 3–5 times increase in muscle mass and strength when compared to transdermal testosterone (Skinner et al., 2018). However, TRT may be contraindicated for many sarcopenic patients with normal blood testosterone levels, as it would cause an abnormal increase in hormonal concentration (McKee et al., 2017; Stangl et al., 2019). Some other AAS, such as Nandrolone Decanoate (ND) and Oxandrolone, demonstrated efficacy in increasing strength and muscle mass in clinical conditions (AIDS, Chronic kidney disease, and COPD), eugonadal older man, and older women (Creutzberg et al., 2003; Grunfeld et al., 2006; Johansen et al., 2006; Sardar et al., 2010; Mavros et al., 2015). There is still no clear evidence about which type of AAS is most effective and suitable for sarcopenia.

The safety of AAS use is still the major obstacle to AAS therapy in the sarcopenic population. In longitudinal non-clinical trial studies, TRT has been shown to reduce the risk of mortality, the incidence of cardiovascular events, and prostate cancer (Wallis et al., 2016; Jasuja et al., 2019; Haider et al., 2020). However, these studies still present some selection bias. AAS and its impact on major outcomes, such as mortality, cardiovascular events, and hospitalization remain to be clarified by future RCTs. The TRT for Assessment of long-term Vascular Events and efficacy Response in hypogonadal men (TRAVERSE) study is an ongoing randomized, double-blind, placebo-controlled, parallel-group, non-inferiority, and multicenter study (Bhasin et al., 2021b). This adequately powered randomized trial with long-term safety of TRT will answer many questions on cardiovascular safety.

## Conclusion

**Figure 1:**
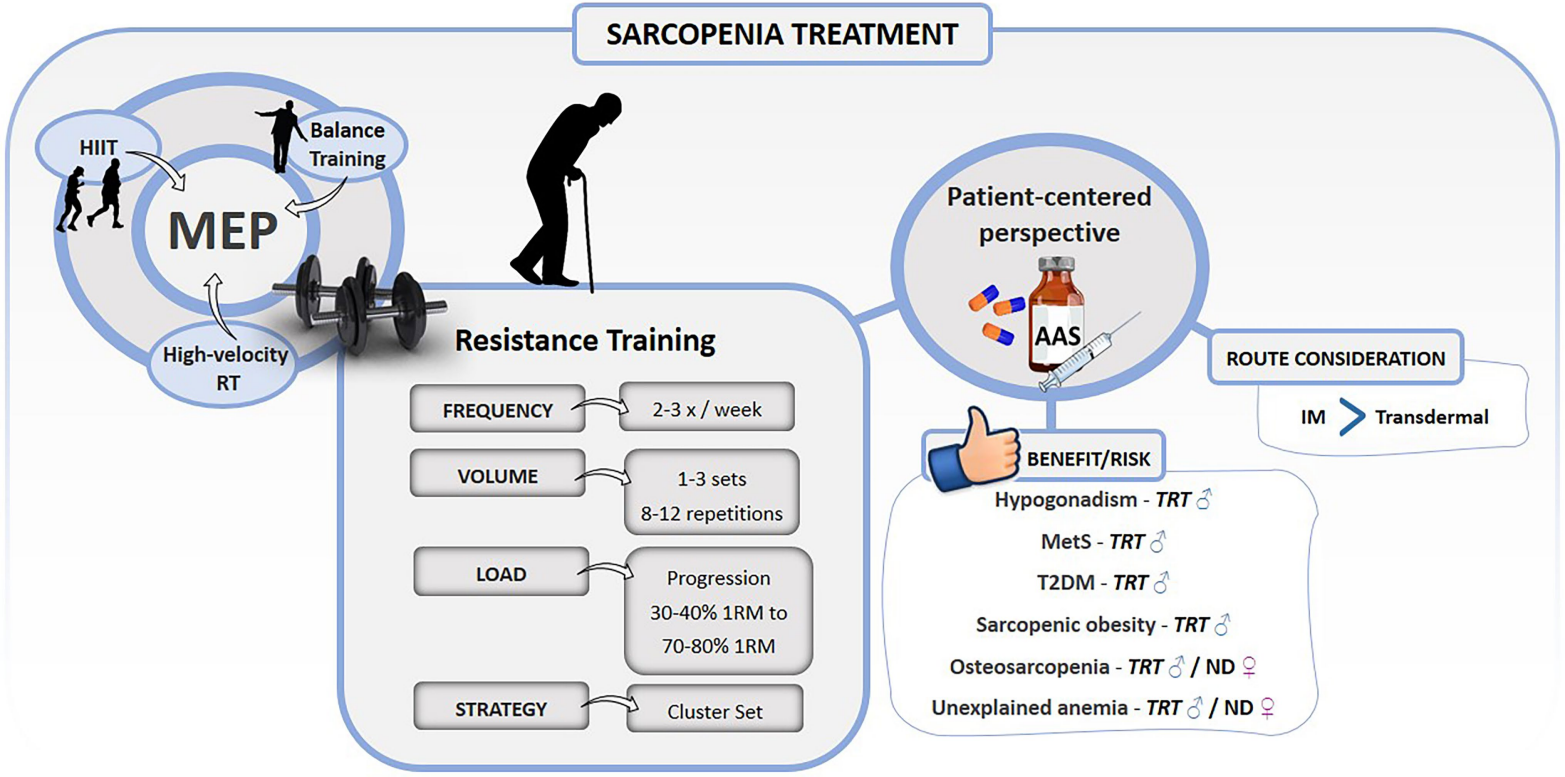
Summary of exercise training and anabolic androgenic steroids recommendations for the treatment of sarcopenia. *1RM*, 1 repetition maximum; *AAS*, anabolic androgenic steroids; *HIIT*, high-intensity interval training; *IM*, intramuscular; *MetS*, metabolic syndrome; *ND*, nandrolone decanoate; *RT*, resistance training; *T2DM*, type 2 diabetes mellitus; *TRT*, testosterone replacement therapy; ♂: men; and ♀: women.

Healthy physical function depends on an intricate relation of physiological factors, which make any pharmacological therapy very unlikely to be the *“magic bullet.”* Exercise represents the main approach that can impact the multi-domain determinants of sarcopenia (physical function and muscle mass). The MEP has shown to be a reliable approach that should be incorporated into the sarcopenic patient's routine, considering their limitations, idiosyncrasies, and comorbidities. Exercise programs must be recommended by health professionals who will evaluate periodically the progression of sarcopenic factors, such as strength, power, physical performance, and muscle mass. Accordingly to individual response to exercise overtime in the aforementioned parameters, exogenous use of AAS may be considered in the case of severe progression of sarcopenia (Figure 1). Despite the suggestive role of TRT in the prevention/treatment of sarcopenia, the exercise training associated with lifestyle changes should be the first-line approach to manage this condition, since there are still inconclusive data on the safety, efficacy, and other drug interaction of long-term AAS usage.

On the other hand, the decision to use AAS should be guided by an evidence-based patient-centric perspective, with the assessment of the potential benefits and risks (Bhasin, 2021). Sarcopenic patients may have other disorders, such as hypogonadism, MetS, obesity, TDM2, osteoporosis, unexplained anemia, and malnutritional status that favor the use of AAS. However, further long-term randomized clinical trials should be carried out to investigate the safety and efficacy of AAS therapy in sarcopenia alone and individuals with other comorbidities. Despite the well-known physiological effects of testosterone, there is no consensus of its use in clinical practice as an adjuvant or main treatment for muscle-loss conditions.

## Author Contributions

HF and LM contributed with the original idea of the article, search of bibliographic references, and elaboration of the manuscript. MS contributed with bibliographic reference research and manuscript elaboration. All authors contributed to the article and approved the submitted version.

## Conflict of Interest

The authors declare that the research was conducted in the absence of any commercial or financial relationships that could be construed as a potential conflict of interest.

## Publisher’s Note

All claims expressed in this article are solely those of the authors and do not necessarily represent those of their affiliated organizations, or those of the publisher, the editors and the reviewers. Any product that may be evaluated in this article, or claim that may be made by its manufacturer, is not guaranteed or endorsed by the publisher.
